# Simplified binomial estimation of human malaria transmission exposure distributions based on hard classification of where and when mosquitoes are caught: statistical applications with off-the-shelf tools

**DOI:** 10.1186/s13071-021-04884-2

**Published:** 2021-08-03

**Authors:** Gerry F. Killeen, April Monroe, Nicodem J. Govella

**Affiliations:** 1 School of Biological, Earth & Environmental Sciences and Environmental Research Institute, University College Cork, Cork, Republic of Ireland; 2 Johns Hopkins Center for Communication Programs, Baltimore, MD USA; 3 Environmental Health and Ecological Sciences Department, Ifakara Health Institute, Ifakara, Tanzania; 4 The School of Life Science and Bioengineering, The Nelson Mandela, African Institutions of Science and Technology, Tengeru, P.O. Box 447, Arusha, United Republic of Tanzania

**Keywords:** Malaria, *Plasmodium*, Arbovirus, Lymphatic filariasis, Mosquito, *Anopheles*, *Culex*, *Aedes*, *Mansonia*, Human–vector interaction, Behaviour

## Abstract

The impacts and limitations of personal protection measures against exposure to vectors of malaria and other mosquito-borne pathogens depend on behavioural interactions between humans and mosquitoes. Therefore, understanding where and when they overlap in time and space is critical. Commonly used approaches for calculating behaviour-adjusted estimates of human exposure distribution deliberately use *soft classification* of where and when people spend their time, to yield nuanced and representative distributions of mean exposure to mosquito bites across entire human populations or population groups. However, these weighted averages rely on aggregating individual-level data to obtain mean human population distributions across the relevant behavioural classes for each time increment, so they cannot be used to test for variation between individuals. Also, these summary outcomes are quite complex functions of the disaggregated data, so they do not match the standard binomial or count distributions to which routine off-the-shelf statistical tools may be confidently applied. Fortunately, the proportions of exposure to mosquito bites that occur while indoors or asleep can also be estimated in a simple binomial fashion, based on *hard classification* of human location over a given time increment, as being either completely indoors or completely outdoors. This simplified binomial approach allows convenient analysis with standard off-the-shelf logistic regression tools, to statistically assess variations between individual humans, human population subsets or vector species. Such simplified binomial estimates of behavioural interactions between humans and mosquitoes should be more widely used for estimating confidence intervals around means of these indicators, comparing different vector populations and human population groups, and assessing the influence of individual behaviour on exposure patterns and infection risk. Also, standard sample size estimation techniques may be readily used to estimate necessary minimum experimental scales and data collection targets for field studies recording these indicators as key outcomes. Sample size calculations for field studies should allow for natural geographic variation and seasonality, taking advantage of rolling cross-sectional designs to survey and re-survey large numbers of separate study locations in a logistically feasible manner.

**Supplementary Information:**

The online version contains supplementary material available at 10.1186/s13071-021-04884-2.

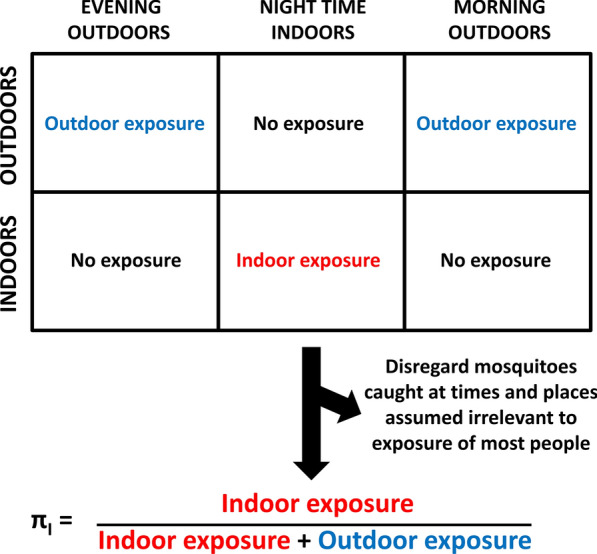

## Background

Assessments of malaria control measures that target human-biting adult mosquitoes require clear understanding of how effectively they protect individual end users and how gaps in personal protection arise [1–4]. Improved understanding of behavioural interactions between humans and mosquitoes, especially where and when they overlap in time and space, is critical to estimating the impact of personal protection measures such as insecticide-treated nets (ITNs) and identification of where and when supplementary vector control tools are needed [1–4]. Similar issues are relevant to personal protection against a variety of other vector-borne pathogens, especially arboviruses such as dengue, Zika and chikungunya carried by mosquito species like *Aedes aegypti* that often bite people while they are active outdoors [5].

While methods for weighting estimates of human exposure to bites from *Anopheles* mosquitoes according to where people spend their time have been available for decades [6], they remain underutilized [1–4, 7]. Crude indoor and outdoor biting rates are still commonly misinterpreted as being indicative of human-vector contact patterns, without any adjustment for the influence of human behaviour or the personal protection effects of ITNs [1–4, 7]. Fortunately, these deficits in current common practice may be readily addressed by relatively straightforward adjustments to data collection and analysis practice [4, 7]. More accurate representations of exposure patterns can be achieved by supplementing mosquito biting activity data with complementary surveys of a small set of human behavioural variables that capture the following over the course of the night: (i) the distribution of human populations indoors and outdoors, (ii) whether they are awake or asleep and (iii) if and when they use an ITN [4, 7]. Important examples of useful indicators that can be calculated with such behaviour-weighted approaches include the proportion of vector bites occurring indoors for an unprotected individual (${\pi }_{I}$) and the proportion of vector bites occurring while asleep for an unprotected individual (${\pi }_{S}$), as well as derived terms like the proportion of all vector bites directly prevented by ITN use or the proportion occurring indoors despite ITN use [4, 7]. If surveyed, estimated and interpreted consistently, these indicators can greatly improve understanding of how malaria transmission persists despite high coverage of prevention measures such as ITNs, how exposure patterns may change as supplementary vector control tools are introduced, and the potential impacts of these new tools [1–4, 7].

The most widely used approaches for calculating behaviour-adjusted estimates of human exposure distribution deliberately use *probabilistic* or *soft classification* to allow for the considerable variability between individual people in terms of where and how they spend their time [4, 7]. The probabilities that any given individual will be indoors or outdoors during a given time increment is estimated as the proportion recorded as doing so through questionnaire or observational surveys of the human population [4, 7]. These probabilities are then used to weight entomological measurements of human exposure to mosquito bites occurring indoors and outdoors, yielding nuanced and representative distributions of mean exposure to biting mosquitoes across entire human populations or population groups [4]. However, these weighted averages rely on aggregating individual-level data to obtain mean human population distributions across the relevant behavioural classes for each time increment. These summary outcomes are therefore quite complex functions of the disaggregated data, so they do not match the standard binomial or count distributions to which routine off-the-shelf statistical tools may be confidently applied. Consequently, testing for variation between individuals, much of which may be associated with epidemiologically important covariates such as age, sex, occupation and housing [7–14], requires advanced Bayesian techniques that are beyond the reach of most field entomologists and epidemiologists.

**Fig. 1:**
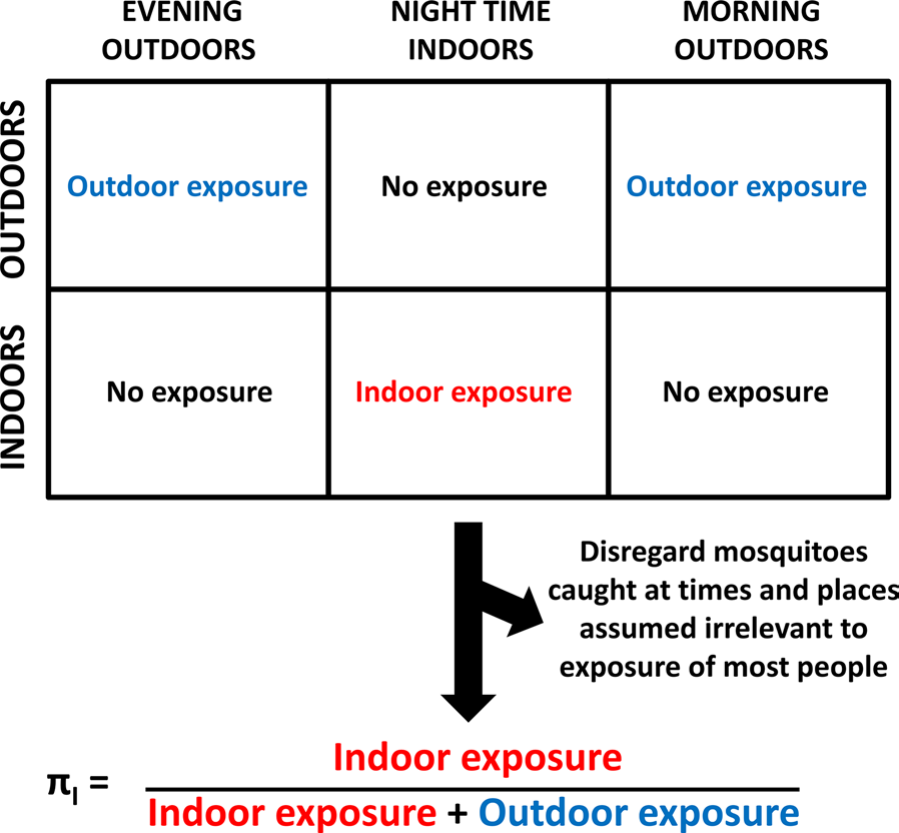
A schematic illustration of how the proportions of human exposure to mosquitoes occurring indoors may be estimated as a simple binomial indicator based on hard classification of where human individuals and populations spend their time [16–21]

Fortunately, the proportions of exposure to mosquito bites that occur indoors or while asleep can also be estimated in a more simplified binomial fashion, based on *hard classification* of human location at a given time increment, as being either completely indoors or completely outdoors [15]. Such clear-cut assignment of humans to either location compartment then allows each mosquito caught attacking a person to be simplistically and unambiguously assigned to one of three categories, on the basis that it was either: (i) caught at a time and place when most people may practically protect themselves by using an ITN, (ii) caught at a time and place when people cannot practically use an ITN or (iii) caught at a time and place assumed to be irrelevant to normal exposure patterns because the majority of people are elsewhere (Fig. 1). This simplification is obviously cruder and less precise because it disregards many valid observations of mosquito-human interactions that occur in the evenings and mornings, when some people are asleep indoors while others are awake outdoors.

However, it does offer the advantage of allowing convenient analysis with standard logistic regression methods, to statistically estimate confidence intervals around means, compare vector species and human population groups and assess the influence of individual behaviour on exposure patterns and malaria risk [16–21] (Additional file 1: Poster S1). Also, because such hard classification techniques allow these key indicators to be calculated in simple binomial form, standard sample size estimation techniques may be readily used to estimate necessary minimum experimental scales and data collection targets required for field studies. While crude hard classification understandably appears to provide less precise estimates, no consistent trend toward under- or overestimation was obvious in a multi-country study encompassing ten different mosquito populations from across Africa [9]. Furthermore, hard and soft classification approaches have different advantages and disadvantages, so both may be included for complementary purposes in a given report [9].

Here, we present a brief explanation and discussion of relatively simple methodological options for applying such hard classification techniques to address common objectives in epidemiological entomology [22, 23]. We place particular emphasis on relatively simple approaches and tools that are accessible to numerate entomologists familiar with widely available, off-the-shelf analytical tools.

## Simplified binomial estimation of human exposure distributions based on hard classification of where and when mosquitoes are caught

Simplified binomial estimation of the proportion of exposure of unprotected individuals to mosquito bites that occurs indoors requires hard classification of human behaviours (Box 1), assuming the night is split into distinct periods, during which all exposure is assumed to occur either entirely indoors or entirely outdoors (Fig. 1). Here, we rely on examples relevant to malaria transmission by predominantly nocturnal vectors, so we use the term *night* to inclusively denote all times at which such *Anopheles* mosquitoes are active, even if that includes daylight hours before dusk and after dawn. For more diurnal vectors of other pathogens such as dengue, chikungunya and Zika, the term *day* may be more appropriate and can include all 24 h of the daily cycle [15] whenever relevant.

The calculations described in Box 1 have already featured in several published applications [15–21] that readers may draw on as illustrative examples when adapting these techniques to their own needs. In the first of these examples [15], it was demonstrated that most human exposure to *Anopheles funestus* and *An. quadriannulatus* in southeastern Zambia occurred indoors in the absence of a protective ITN (${\pi }_{I,u}$ ≥ 0.97), because these mosquito species are most active at times of the night when humans are indoors (${P}_{FL,I}$ ≥ 0.90). Note, however, that if one only accounts for the behaviour of the mosquitoes, as observed attacking participants in human landing catches who artificially spend equal amounts of time indoors and outdoors for the purpose of such experiments, these two mosquito populations had no apparent preference for feeding indoors (${P}_{I}$ ≈ 0.5) [15]. Subsequent pooled analysis of similar data from distinct sites scattered across Africa revealed similar patterns [16], confirming that the most important vectors on the continent are not innately endophagic in the strict sense but rather highly nocturnal. Therefore, it was concluded that high proportions of human exposure to malaria transmission have historically occurred indoors because that is where most people sleep at night [1, 3, 23, 25]. Further applications of such simplified binary formats for behavioural interaction indicators include demonstrating statistically significant changes in human exposure distributions following scale up of ITNs [17, 18, 21].

Beyond comparing vector species and human population groups, these simplified binary behavioural interaction indicators (Box 1) can also be used to assess the influence of variable, often idiosyncratic individual behaviours on exposure patterns and malaria risk [16–21]. Whenever possible, human behaviour data should therefore be collected in a disaggregated format that is linkable to individual human study participants, so that the epidemiological importance of differences between individuals and population subgroups can be formally assessed [4] with statistical contrasts using standard logistic regression models and off-the-shelf software [19, 20]. For ease of application to readers own data sets, a Microsoft Excel® template for calculating individual-level estimates of ${\pi }_{I}$ and ${\pi }_{S}$ is provided and populated with a sample of anonymized data from previous large-scale cross-sectional surveys [20] for illustrative purposes (Additional file 2: Dataset S1). The additional insight that may be obtained from disaggregated data with such individual-level calculations is exemplified by comparing the in-depth epidemiological analyses reported along with the original version of this template [20] with the much broader, population-wide mean overview obtained through the preceding entomological report [26].

Box 1 Mathematical description of how simplified binomial estimates of human exposure distributions may be calculated based on hard classification of where and when mosquitoes are caught First, one must divide the night, or entire 24-h day if needs be, into hour-long survey time intervals (*t* = 0, 1, 2, 3…23). These hour-long intervals are then conceptually grouped into three distinct periods, during which all exposure is assumed to occur either entirely indoors or entirely outdoors (Fig. 1). Note, however, that even previous surveys of where and when people spent their time in the same context may not necessarily remain fully representative going forward, so it may be unwise to assume population-wide mean cut-off points that separate these three distinct periods *a priori*. Also note that one of the most important applications of this approach is to enable analysis of the behavioural variations between individuals in a population, so it is essential to collect both human and mosquito behaviour data in fully disaggregated form at time intervals of ≤ 1 h in duration. Once the data have been collected in appropriately disaggregated hour-by-hour format, the middle period of the night when all exposure is assumed to occur while asleep indoors (Fig. 1) is defined as beginning at the first ($F,I$) and ending at the last ($L,I$) of the surveyed hour-long time (*t*) intervals when either (i) a *human individual* reported being indoors (${I}_{t}=1$) or (ii) the *majority of a human population* were indoors (${I}_{t}>0.5$), because they reported that they had already entered their houses for the night and had not yet left for the day ($F,I\le t\le L,I$). The remaining periods of the night, before ($t<F,I$) and after ($t>L,I$) this interval, correspond to periods when either (i) an individual or (ii) most people in a population are outdoors (${I}_{t}<0.5$). Correspondingly, the proportion of human exposure for unprotected (*u*) non-users of ITNs that occurs indoors (${\pi }_{I,u}$) may be approximately calculated by dividing the number of mosquitoes caught indoors during the period that most people are indoors (${N}_{I}$) by itself plus the number of mosquitoes caught outdoors (${N}_{o}$) outside of that period [15, 17, 18]:

1 $$\pi_{I,u} = \mathop \sum \limits_{t = F,I}^{L,I} \left[ {N_{I,t} } \right]/\left( {\mathop \sum \limits_{t = 0}^{F,I - 1} \left[ {N_{O,t} } \right] + \mathop \sum \limits_{t = F,I}^{L,I} \left[ {N_{I,t} } \right] + \mathop \sum \limits_{t = L,I + 1}^{23} \left[ {N_{O,t} } \right]} \right)$$

The equivalent binomial calculations may also be made for the proportion of exposure to mosquito bites of unprotected individuals which occurs while asleep (${\pi }_{S,u}$), using the first ($F,S$) and last ($L,S$) hour-long intervals when either (i) an individual (${S}_{t}=1$) or (11) most people in a population were asleep (${S}_{t}>0.5$):

2 $$\pi_{S,u} = \mathop \sum \limits_{t = F,S}^{L,S} \left[ {N_{I,t} } \right]/\left( {\mathop \sum \limits_{t = 0}^{F,S - 1} \left[ {N_{O,t} } \right] + \mathop \sum \limits_{t = F,S}^{L,S} \left[ {N_{I,t} } \right] + \mathop \sum \limits_{t = L,S + 1}^{23} \left[ {N_{O,t} } \right]} \right)$$

To more clearly interpret the ${\pi }_{I,u}$ and ${\pi }_{S,u}$ estimates obtained [16], the two following underlying determinants of these outcomes may also be calculated [15, 17, 18]. The propensity of vectors to feed indoors is reflected in the proportion of mosquitoes captured indoors (${P}_{I}$):

3 $$P_{I} = \mathop \sum \limits_{t = 0}^{23} \left[ {N_{I,t} } \right]/\mathop \sum \limits_{t = 0}^{23} \left[ {N_{I,t} + N_{O,t} } \right]$$

The propensity of vectors to feed at times when people are indoors is reflected in the proportion of all mosquitoes caught that were captured during hours when the majority of people were indoors (${P}_{FL,I}$):

4 $${P}_{{{FL},{I}}} = \mathop \sum \limits_{{{t} = {F},{I}}}^{{{L},{I}}} \left[ {{N}_{{{I},{t}}} + {N}_{{{O},{t}}} } \right]/\mathop \sum \limits_{{{t} = 0}}^{23} \left[ {{N}_{{{I},{t}}} + {N}_{{{O},{t}}} } \right]$$

Similar calculations can be made for the propensity of vectors to feed at times when people are asleep (${P}_{FL,S}$), which may paint a more nuanced and accurate picture of the underlying behavioural drivers of human exposure distribution in some contexts [20, 24]:

5 $${{P}_{FL,S}}=\underset{t=F,S}{\overset{L,S}{\mathop \sum }}\,\left[ {{N}_{I,t}}+{{N}_{O,t}} \right]/\underset{t=0}{\overset{23}{\mathop \sum }}\,\left[ {{N}_{I,t}}+{{N}_{O,t}} \right]$$

All these indicators of propensity of vectors to feed indoors (${P}_{I}$) and during the night time hours predominantly spent indoors (${P}_{FL,I}$, referred to as nocturnality [17] or nocturnal biting [18] in previous publications) or asleep (${P}_{FL,S}$) may be formally tested for vector preference (${P}_{I}$,${P}_{FL,I} \, \text{or} \, {P}_{FL}\ne 0.5$) in terms of the significance of the differences of these estimates from the null hypothesis (${P}_{i}$,${P}_{FL,I} \, \text{or} \, {P}_{FL}=0.5$).

## Simplified sample size calculations for studies surveying the proportions of human exposure to malaria vectors occurring indoors or while asleep

**Fig. 2:**
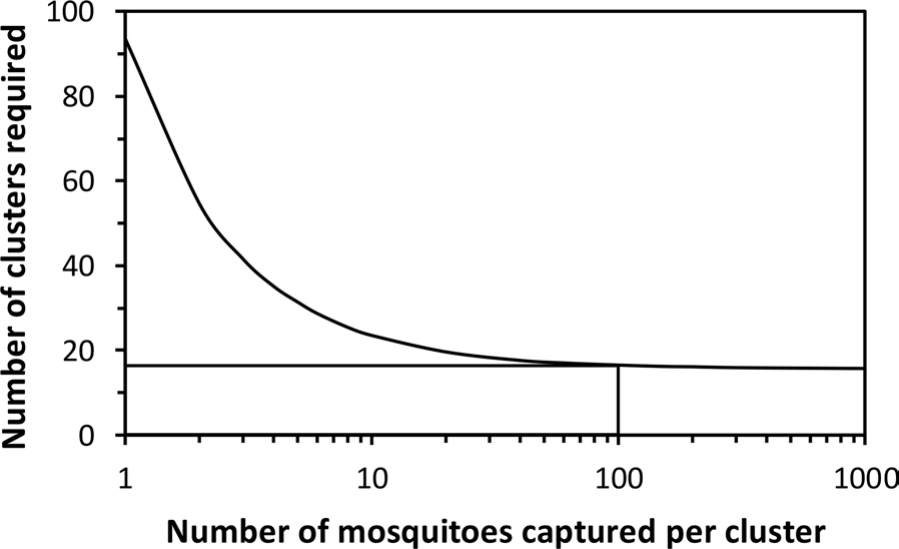
The predicted relationship between the number of mosquitoes caught per cluster and the number of village-scale population clusters required to achieve 80% power if all other assumed parameters are exactly as described in Box 2. Note that in the field of epidemiology, the simple term *cluster* usually refers a geographically distinct, but often demographically defined, unit of observation that may be considered independent in the statistical sense [27]

Because these hard classification techniques allow the proportions of human biting exposure occurring indoors (${\pi }_{I,u}$) and while asleep (${\pi }_{S,u}$) to be calculated in simple binomial form, it is also possible to apply standard sample size estimation techniques [27] to plan necessary minimum experimental scales and data collection targets for field studies (Fig. 2). To enable sample size estimation with the most intuitive and readily accessible statistical tools, here an example is provided (Box 2) that adapts well-established simple deterministic statistical models, originally formulated for cluster-randomized trials with disease infection prevalence as the primary binomial outcome [27]. Of course, more advanced, simulation-based stochastic approaches may also be applied to sample size calculations for surveying these same binary indicators [28], and these more intricate techniques may be more rigorous and appropriate for teams with sufficient analytical capacity. However, relevant analytical capacity remains underdeveloped for malaria generally, and entomology in particular, in endemic tropical countries [3]. Therefore, the simple but widely accepted deterministic models [27] and calculation tools (Additional file 3: Dataset S2) used in Box 2 may represent an accessible and practical alternative for teams at an earlier stage of analytical capacity development.

Note that while many investigators may not need to implement surveys across such extensive scales, they should nevertheless allow for the fact that some natural variation of *π*_*I,u*_ and *π*_*S,u*_ is likely to occur among different sub-villages, villages or other geographic subunits/population clusters far enough apart to be considered independent units of observation. Furthermore, such natural spatial variation may be further exaggerated by natural seasonality, which may also be of interest in its own right. Therefore, sample size calculations should always allow for clustering and covariance within clusters, as in the example provided in Box 2. Note that it may not be logistically feasible to survey all different study locations simultaneously, especially on scales of entire districts or countries. Rolling cross-sectional designs that survey clusters sequentially (e.g. [14, 31]), rather than at the same time, may therefore be necessary to survey and resurvey the large numbers of distinct locations sometimes required to achieve sufficient power (Fig. 2).

Box 2 An example of a sample size calculation for a nationally representative survey of the proportion of human exposure to malaria vectors occurring indoors in the United Republic of Tanzania A nationally representative survey of key mosquito and human behaviours is underway in Tanzania, for which one of the two key primary outcomes is the proportion of human exposure occurring indoors in the absence of an ITN (*π*_*I,u*_). For practical logistics reasons, this very large-scale study has a rolling cross-sectional design, enabling each of the village-scale independent units of observation (referred to as *clusters* in epidemiological terminology [27]) to be feasibly surveyed in a sequential manner once per year. This is an observational study, so the groups of clusters being contrasted will be eco-epidemiological strata rather than the interventions groups allocated in an equivalent experimentally controlled trial. For example, one *a priori* hypothesis of this particular study is that high humidity may enable mosquitoes to feed earlier in the evening and later in the morning without becoming desiccated, leading to lower values of *π*_*I,u*_ and attenuated impacts of ITNs. The simplest sample size calculation that may be carried out therefore assumes that all the surveyed clusters will be ranked *post hoc* based on humidity measurements and then split into two quantiles, namely the most humid half of the surveyed clusters versus the least humid half. Based on the following assumed input parameters, and using Eq. 4 from Hayes and Bennett’s widely cited article [27], the minimum number of village-scale survey clusters (*c*) required to achieve 80% power for contrasts between two such groups was estimated to be 16, assuming a minimum of 100 mosquitoes are caught in each village-scale cluster (Fig. 2, Additional file 3: Dataset S2) at times and places when most humans are exposed (Fig. 1). The calculations underpinning Fig. 2 and detailed in Additional file 3: Dataset S2 are explained as follows. For the outcome of interest, namely the proportion of human exposure occurring indoors (*π*_*I,u*_), the following contrast between two previously surveyed settings was assumed to be representative of the level of variation that could be epidemiologically relevant, because persistent residual malaria transmission depends more directly on the relative size of gaps in *de facto* protective coverage (*1-C*_*p*_, where *C*_*p*_ = *π*_*I,u*_* C* [29]) than upon protective coverage *per se* (*C*_*p*_) [24]): One rural Tanzanian setting where 90% of exposure to *Anopheles gambiae* was estimated to occur indoors and another where 73% of exposure to the same species occurred indoors in an urban setting [26], so *π*_*I,u,1*_ = 0.90 and *π*_*I,u,2*_ = 0.73, respectively. While this may sound like a relatively minor difference, when looked at in terms of a potential gap in protective coverage (*1-C*_*p*_) [24], it represents almost a tripling of the fraction of exposure occurring outdoors, from 10 to 27%. Based on experience and results from limited studies in Tanzania, plus those reported from several rural settings across Africa [16], only a modest coefficient of variation between clusters (*k*) of 0.20 was assumed. Additionally, a worst case scenario was assumed in terms of mosquito abundance and capture success, with the number of mosquitoes in each cluster (generally a village or sub-village in most epidemiological or entomological studies, and specifically the former in this particular study) collected at times and places when human exposure may occur (Fig. 1) assumed to be relatively low (*n* = 100 mosquitoes per cluster). As illustrated in Fig. 2 and explained as follows, a total of 16 clusters per stratum are expected to yield sufficient power to test for between-stratum differences in *π*_*I,u*_ of the magnitude described above, even if far less than 100 mosquitoes are caught in some of the surveyed clusters.

Even with a tenfold increase beyond the within-cluster sample size target for this study, up to 1000 mosquitoes per village is expected to yield very little improvement in overall power (Fig. 2) because doing so does not increase true replication in the strict sense [27, 30]. This is because individual mosquitoes caught within a surveyed cluster merely represent binary observations on multiple samples from within that same cluster, rather than truly independent observations from distinct cluster-level replicates *per se*. Mosquitoes sampled from the same population in the same village, experiencing the same environmental conditions at the time when it is surveyed, are obviously expected to behave more similarly to each other than to mosquitoes of the same species collected from another village in a different part of the country, where the environmental conditions may be very different, especially if it is visited at a different time of the year. Unless such intra-cluster correlation between sampled observations is allowed for, *pseudoreplication* renders invalid any subsequent analyses that erroneously treat them as independent observations [27, 30]. The deterministic predictive models of Hayes and Bennett therefore conservatively account for the expected similarities between observed individuals in each cluster in the simplest fashion possible, with a single within-cluster variance parameter [27]. Based on the envisaged protocol, the number of mosquitoes expected per surveyed cluster should therefore be considered as a single aggregate total for parameterizing *n* in Additional file 3: Dataset S2, regardless of how many houses are to be sampled or how many nights they are sampled over. For binary outcomes with binomial distributions, remarkably small samples can yield quite precise estimates of the mean for any given unit of observation, so even as few as 100 mosquitoes per cluster may be expected to achieve essentially the maximum possible power, thus minimizing cluster replication requirements (Fig. 2). Recognizing that excessive within-cluster sampling, to capture hundreds or even thousands of mosquitoes per village, adds negligible power to appropriate statistical tests (Fig. 2) also has encouraging implications for the robustness of the study design to seasonal or location-specific scarcities of mosquitoes: Even if < 100 mosquitoes are caught in each cluster, this is expected to have only a modest effect on the power of the study, so long as at least ten are caught (Fig. 2) in the times and places that matter (Fig. 1). Thus, even if this study falls short of this *a priori* target of 100 mosquitoes in some of the villages surveyed, this is not a major cause for concern or for alteration of the study protocol.

## Conclusions

Simplified binomial estimates of the proportions of exposure to mosquito bites that occur indoors or while asleep, based on *hard classification* of human location at a given time, allow convenient statistical analysis to estimate confidence intervals around means, compare vector species and human population groups, and assess the influence of individual behaviour on exposure patterns and malaria risk. Also, because such hard classification techniques allow these key indicators to be calculated in simple binomial form, standard sample size estimation techniques may be readily used to plan *a priori* the necessary experimental scales and data collection targets required for field studies. Sample size calculations for field studies should allow for natural geographic variation and seasonality, taking advantage of rolling cross-sectional designs to survey and re-survey's large numbers of separate study locations in a logistically feasible manner.

## Supplementary Information

**Additional file 1: Poster S1.** A poster illustrating the basic principles of simple binomial *hard classification* of where and when biting mosquitoes are caught, plus examples of applications of this approach using routine off-the-shelf statistical tools to compare mosquito populations and human population groups or calculate suitable sample sizes for large-scale field surveys.

**Additional file 2: Dataset S1.** An Excel® spreadsheet template for calculating individual-level estimates for the proportion of exposure of bites by *Anopheles gambiae* (*s.l.*) that would occur indoors or while asleep in the absence of any protective interventions, such as window screens or bed nets, in the Tanzanian city of Dar es Salaam [20], as illustrated in Additional file 1: Poster S1. This example is populated with an anonymized sample of questionnaire data describing the times residents reported having gone indoors for the evening, gone to sleep for the night, woke up in the morning and left the house in the morning, as well as published patterns of vector biting activity as measured by human landing catch in parts of Dar es Salaam with vector densities that were high enough to measure [20].

**Additional file 3: Dataset S2.** An example of a sample size calculation for a nationally representative survey of the proportion of human exposure to malaria vectors occurring indoors in the United Republic of Tanzania, using an Excel® spreadsheet template to apply Eq. 4 of the classic paper by Hayes and Bennett [27], as explained in Box 2 and illustrated in Fig. 2 and Additional file 1: Poster S1.

## Data Availability

All relevant equations, calculation tools and sample data are included as additional files.

## Abbreviation

ITNs: Insecticide-treated nets
