# Supplementary material for: Simplified binomial estimation of human malaria transmission exposure distributions based on hard classification of where and when mosquitoes are caught: statistical applications with off-the-shelf tools
Source: Parasit Vectors. 2021 Aug 3;14:384. doi: 10.1186/s13071-021-04884-2 (PMC8336365; doi:10.1186/s13071-021-04884-2)

Simplified binomial estimation of human malaria transmission exposure distributions based on hard classification of where and when mosquitoes are caught: Statistical applications with off-the-shelf tools

Gerry F. Killeen[1], April Monroe[2], Nicodem J. Govella[3,4]

[1] School of Biological, Earth & Environmental Sciences and Environmental Research Institute, University College Cork, Cork, Republic of Ireland; [2] Johns Hopkins Center for Communication Programs, Baltimore, Maryland, United States of America; [3] Environmental Health and Ecological Sciences Department, Ifakara Health Institute, Ifakara, Tanzania; [4] The School of Life Science and Bioengineering, The Nelson Mandela, African Institutions of Science and Technology, P.O. BOX 447, Tengeru, Arusha, United Republic of Tanzania

Simplified hard classification to yield binary indicators of exposure distribution

	EVENING OUTDOORS	NIGHT TIME INDOORS	MORNING OUTDOORS
OUTDOORS	Outdoor exposure	No exposure	Outdoor exposure
INDOORS	No exposure	Indoor exposure	No exposure

Disregard mosquitoes caught at times and places assumed irrelevant to exposure of most people

$$\pi_I = \frac{\text{Indoor exposure}}{\text{Indoor exposure} + \text{Outdoor exposure}}$$

Insight: The proportions of exposure to mosquito bites that occur while indoors or asleep can be estimated in a simple binomial fashion, based on *hard classification* of human location over a given time increment. This simplified binomial approach allows convenient analysis with standard off-the-shelf logistic regression tools to statistically assess variations between individual humans, human population subsets or vector species.

Conclusions: Simplified binomial estimates of behavioural interactions between humans and mosquitoes should be more widely used for estimating confidence intervals around means of these indicators, comparing vector species and human population groups, and assessing the influence of individual behaviour on exposure patterns and malaria risk. Also, standard sample size estimation techniques may be readily used to estimate necessary minimum experimental scales and data collection targets for field studies recording these indicators as key outcomes.

Further reading: (1) Killeen GF et al (2018) Entomological surveillance as a cornerstone of malaria elimination: a critical appraisal. In: *Towards Malaria Elimination-A Leap Forward*. Eds Dev V, Manguin S; InTech: 403-429; (2) Monroe A et al (2020) Methods and indicators for measuring patterns of human exposure to malaria vectors. Malar J. 19: 207; (3) Huho et al (2013) Consistently high estimates for the proportion of human exposure to malaria vector populations occurring indoors in rural Africa 43: 235; (4) Msellemu et al (2016) The epidemiology of residual *Plasmodium falciparum* malaria transmission and infection burden in an African city with high coverage of multiple vector control measures. 15: 288

Individual-level estimates for humans within populations and population groups

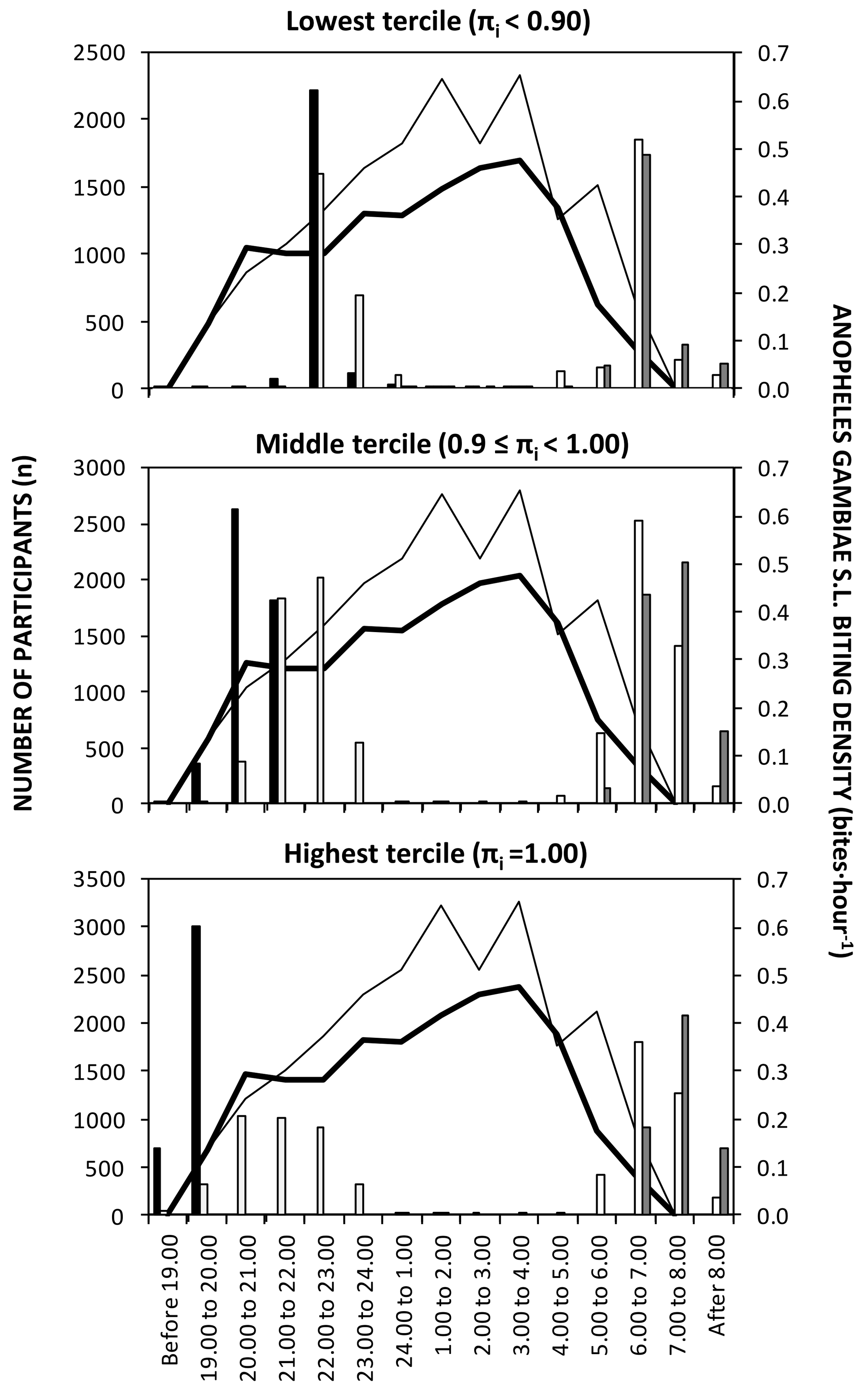

Times at which individuals interviewed during cross-sectional household surveys in Dar es Salaam, Tanzania reported having gone indoors for the evening, gone to bed for the evening, gotten out of bed in the morning and left the house in the morning, stratified by derived individual estimates for the proportion of exposure to *An. gambiae* mosquito bites that would occur indoors in the absence of a bed net ($\pi_{I,u}$ see additional file 2 and reference 4 for details)

Compare and contrast mosquito populations

Estimates for proportions of mosquitoes caught indoors ($P_{I,u}$), proportion of mosquitoes caught between the first and last hour when most humans were indoors ($P_{FL,I}$), and proportion of human exposure to mosquito bites occurring indoors ($\pi_{I,u}$) for *Anopheles gambiae s.l.* at six sites across Africa (See reference 3 for details)

Site	*n*	P_I (95% CI)	*P*-value	$P_{FL,I}$ (95% CI)	*P*-value	*n*	$\pi_{I,u}$ (95% CI)	*P*
	Overall effect of site:		<0.001	Overall effect of site:	<0.001	Overall effect of site:		<0.001
Rarieda	337	0.54 (0.48, 0.59)	0.174	0.78 (0.73, 0.82)	<0.001	187	0.79 (0.72, 0.84)	<0.001
Luangwa	638	0.63 (0.60, 0.67)	<0.001	0.84 (0.81, 0.87)	<0.001	380	0.90 (0.87, 0.93)	<0.001
Rufiji	102	0.46 (0.36, 0.56)	0.429	0.99 (0.93, 1.000)	<0.001	48	0.98 (0.87, 1.00)	<0.001
Ulanga	320	0.40 (0.34, 0.45)	<0.001	0.91 (0.88, 0.94)	<0.001	127	0.89 (0.82, 0.93)	<0.001
Oubritenga	1377	0.57 (0.55, 0.60)	<0.001	1.00		791	1.00	
Kourweogo	1019	0.62 (0.59, 0.65)	<0.001	1.00		637	1.00	

Sample size calculations for national surveys

See Box 2 and additional file 3 for details

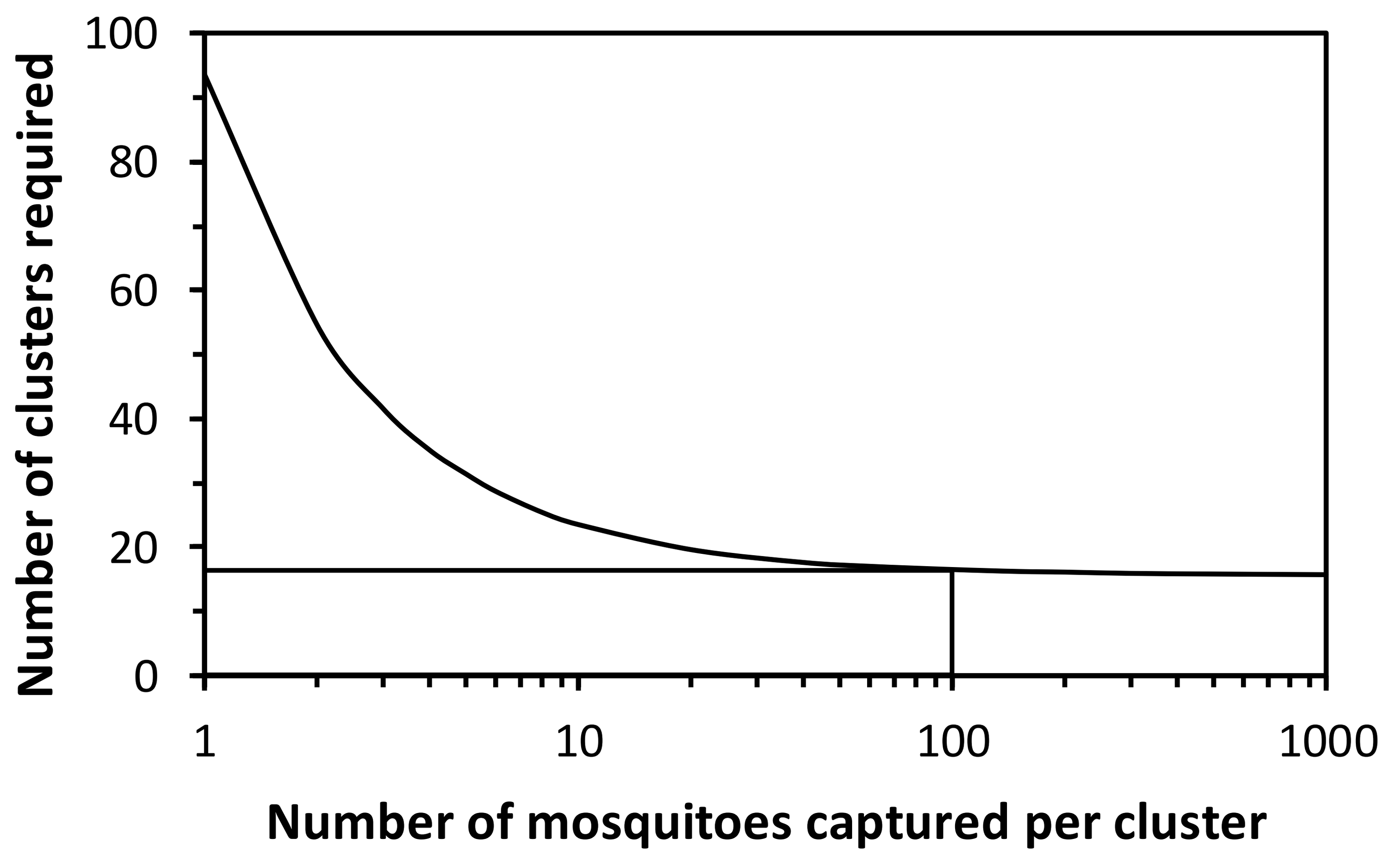

Supplement: Supplementary file 1 — Additional file 1: Poster S1. A poster illustrating the basic principles of simple binomial hard classification of where and when biting mosquitoes are caught, plus examples of applications of this approach using routine off-the-shelf statistical tools to compare mosquito populations and human population groups or calculate suitable sample sizes for large-scale field surveys. [file 13071_2021_4884_MOESM1_ESM.pdf]
